# How often is a work-up for *Legionella *pursued in patients with pneumonia? A retrospective study

**DOI:** 10.1186/1471-2334-11-237

**Published:** 2011-09-07

**Authors:** Brian Hollenbeck, Irene Dupont, Leonard A Mermel

**Affiliations:** 1 Department of Medicine, Rhode Island Hospital and The Warren Alpert Medical School of Brown University, 593 Eddy Street, Providence, RI 02903 USA; 2 Department of Epidemiology and Infection Control, Rhode Island Hospital, 593 Eddy Street, Providence RI 02903 USA; 3 Division of Infectious Diseases, Rhode Island Hospital, 593 Eddy Street, Providence, RI 02903 USA

**Keywords:** *Legionella*, Legionnaires' disease, pneumonia

## Abstract

**Background:**

It is unclear how often patients with pneumonia are assessed for *Legionella *in endemic areas. Additionally, the sensitivity of the IDSA/ATS criteria for recommended *Legionella *testing is undefined.

**Methods:**

We performed a single-center, retrospective study of patients diagnosed with *Legionella *pneumonia at our hospital to determine: 1) how often *Legionella *diagnostic testing is obtained on patients with pneumonia at the time of hospitalization or when pneumonia developed during hospitalization; and 2) how often patient's with *Legionella *pneumonia met at least one of the five criteria in the IDSA/ATS guidelines recommending a work-up for *Legionella*. Patients with *Legionella *pneumonia were identified using an infection control software program. Medical records of these patients were then reviewed.

**Results:**

Thirty-five percent of patients with a discharge diagnosis of pneumonia had *Legionella *urine antigen testing and/or a *Legionella *culture performed. Forty-four percent of patients who had a bronchoscopic specimen sent for microbiologic testing had a *Legionella *culture performed on the bronchoscopic specimen and/or *Legionella *urine antigen testing. Of 37 adult patients with *Legionella *pneumonia, 22 (59%) met the IDSA-ATS criteria recommending *Legionella *testing.

**Conclusion:**

Following current recommendations for *Legionella *testing missed 41% of *Legionella *cases in adults in our single-center study. A work-up for *Legionella *(i.e., urine antigen test and/or culture) was performed in less than half of patients who have a bronchoscopic specimen sent for microbiologic testing.

## Background

In the Infectious Diseases Society of America (IDSA) and American Thoracic Society (ATS) community-acquired pneumonia guidelines [1], *Legionella pneumophila *urine antigen testing is recommended for patients with any of the following: severe pneumonia requiring intensive care unit (ICU) admission, failure of outpatient antibiotics, active alcohol abuse, history of travel within the previous two weeks, or pleural effusion. For every patient with community-acquired pneumonia, empiric treatment of *Legionella *is recommended using a macrolide or respiratory fluoroquinolone [1]. However, making a diagnosis of *Legionella *allows the practitioner to more accurately define the choice and duration of antimicrobial therapy and such testing assists public health officials in detecting *Legionella *outbreaks in community and healthcare settings.

The aims of this study were to determine: 1) how often *Legionella *diagnostic testing is obtained by physicians managing all patients with pneumonia; 2) how often *Legionella *testing is obtained for patients who have an heightened acuity of illness prompting bronchoscopic evaluation as a diagnostic test for possible pneumonia; and 3) the sensitivity of current IDSA/ATS guidelines for recommending *Legionella *testing in patients with pneumonia.

## Methods

The TII ECLYPSIS software program (Chicago, IL), which identifies specific ICD-9 codes and billing data, was used to retrospectively determine the number of patients with a primary or secondary diagnosis of pneumonia and to determine the number of patients who had a bronchoscopy and a bronchoscopic specimen sent for microbiologic testing between October 1, 2007 and March 31, 2009. The same software program was used to identify the number of patients who had urine *Legionella *antigen testing (BinaxNow, Inverness Medical Professional Diagnostics, Princeton, NJ) or who had a *Legionella *culture using standard microbiological methods [2].

The TheraDoc^® ^software program (Hospira, Lake Forest, IL) was used by the Department of Epidemiology and Infection Control at Rhode Island Hospital to prospectively identify patients at least 18 years of age with community or hospital-acquired *Legionella *pneumonia between January 1, 2005 and December 31, 2009 in our 719 bed tertiary care, university-affiliated hospital in Southern New England. For the purposes of this study, we retrospectively assessed this data and performed chart reviews as noted below. Each patient was included only once; incarcerated patients were excluded due to IRB restrictions.

Chart reviews were retrospectively performed by one of the authors (BH) to determine demographic, laboratory, and clinical features of each *Legionella *case, including the five criteria for *Legionella *testing noted in the IDSA/ATS guideline as follows: history of alcohol abuse, recent travel within 2 weeks, pleural effusion upon admission, admission to an intensive care unit for pneumonia, and failure of outpatient antibiotics. Both electronic and paper charts were reviewed in a systematic fashion. Hyponatremia was defined as sodium < 130 mEq/L. This study was approved by the Rhode Island Hospital IRB committee (IRB Registration # 00000396). All authors had full access to all the data in the study and take responsibility for the integrity of the data and accuracy of the data analysis.

## Results

There were 3,982 patients of any age with a primary or secondary diagnosis of pneumonia between October 1, 2007 and March 31, 2009. Of these, 1,406 patients (35%) had a *Legionella *urine antigen test and/or *Legionella *culture as follows: 1251 had a urine antigen test alone; 49 had a *Legionella *culture alone; and 106 had both a *Legionella *culture and urine antigen test. During the same time period, 626 patients underwent bronchoscopy and had bronchoscopic specimens sent to the microbiology laboratory for culture, 277 (44%) of whom had *Legionella *testing done as follows: 122 had a *Legionella *culture alone; 31 had a urine antigen test alone; and 124 had both a *Legionella *culture and urine antigen test.

Forty-one adult patients with *Legionella *pneumonia were identified between January 1, 2005 and December 31, 2009, 37 of which were included in the study (Table 1). Reasons for exclusion were as follows: under 18 years of age (2 cases); incarcerated patient (1 case); and *Legionella *pneumonia was diagnosed and treated at an outside hospital but the *Legionella *urine antigen test remained positive when retested at admission to our hospital (1 case). There were 6, 5, 5, 12, and 9 *Legionella *cases in 2005 through 2009, respectively. All 37 cases were *L. pneumophila *serogroup 1. Thirty-six cases were diagnosed by urine antigen testing alone; one case had a positive urine antigen test and a positive *Legionella *culture from a bronchoscopic specimen. Thirty-three of the 37 cases (89%) met CDC surveillance criteria for community-acquired Legionnaires' disease; the remaining four cases (11%) met criteria for possible hospital-acquired pneumonia. All 4 patients with possible hospital-acquired *Legionella *pneumonia were immunosuppressed. Twenty-two of 37 cases (59%) met at least one of the five IDSA/ATS criteria recommending *Legionella *testing as follows: 14 (38%) were admitted to an ICU; 7 (19%) had a pleural effusion on initial chest radiograph; 6 (16%) had a history of alcohol abuse; 4 (11%) failed outpatient antibiotics prior to admission; and 1 (3%) had a history of recent travel. Among the 37 cases, the most common findings were: immunocompromised status, 24 cases (65%); hyponatremia, 24 cases (65%); diarrhea 14 cases (38%), and ICU admission, 14 cases (38%). Chart review revealed that liver function testing was not performed in 8 patients and presence or absence of diarrhea was not recorded in 2 patients.

**Table 1:** Characteristics of Adult Patients with *Legionella *Pneumonia, 2005-2009

	Patients with Legionella that met IDSA/ATS criteria for Legionella testing	Patients with Legionella that did not meet IDSA/ATS criteria for Legionella testing	All patients with Legionella pneumonia
Male	15	9	24 (65%)
Recent travel	1	0	1 (3%)
History of alcohol abuse	6	0	6 (16%)
Anti-TNF therapy	1	1	2 (5%)
Daily steroid use	0	1	1 (3%)
Cancer	4	4	8 (22%)
Diabetes	5	6	11 (29%)
COPD	5	4	9 (24%)
Solid-organ transplant recipient	0	1	1 (3%)
HIV infection	1	0	1 (3%)
Receipt of antibiotics prior to hospitalization	4	0	4 (11%)
Pleural effusion present at hospital admission	7	0	7 (19%)
ICU admission	14	0	14 (38%)
Abnormal liver function tests	15	5	20 (54%)
Hyponatremia (Sodium < 130 mEq/L)	9	4	13 (35%)
Crude mortality	6	0	6 (16%)

SD, standard deviation; TNF, tumor necrosis factor; COPD, chronic obstructive pulmonary disease; abnormal liver function test defined as above reference range; Crude mortality defined as in-hospital death or discharge to hospice care with impending death.

## Discussion

Thirty-five percent of patients with a discharge diagnosis of pneumonia between October 1, 2007 and March 31, 2009 were tested for *Legionella*. Of patients whose acuity of illness led to the need for bronchoscopy and who had a bronchoscopic specimen sent for microbiologic testing, 44% had *Legionella *testing by urine antigen or culture [3]. These results suggest that available methodologies to diagnose *Legionella *may be underutilized in regions of the USA where *Legionella *is endemic.

Of 37 cases of Legionnaires' disease diagnosed in our hospital over 5 years, 22 (59%) met IDSA/ATS criteria for *Legionella *testing. Thus, 41% of *Legionella *cases may have gone undiagnosed if testing was only done according to the IDSA/ATS recommendations. Of the 5 clinical features recommended by IDSA/ATS guidelines, admission to ICU and the presence of a pleural effusion were most helpful in identifying patients with *Legionella *pneumonia. The low number of patients presenting with antibiotic failure may reflect the widespread use of macrolides and quinolones by primary care providers.

Nearly all of our cases were community-acquired and all cases were *L. pneumophila *serogroup 1. Thirty-six of our 37 cases were diagnosed solely by urine antigen testing. This suggests that cases of Legionnaires' disease due to other serogroups may have been missed because *Legionella *culture was underutilized. The CDC estimates 2.5 to 5.8 *Legionella *cases/100,000 population yearly in the USA [4,5]. In Rhode Island, epidemiologic data on Legionnaires' disease are available from 2004 to 2008 [6], during which time the annual incidence ranged from 2.0 to 4.7 cases/100,000 population. There was a trend towards an increasing incidence of *Legionella *pneumonia in Rhode Island during this time period similar to national data suggesting increasing incidence of Legionnaires' disease from 1990 to 2005, with the most pronounced rise in noted in the Northeastern USA [7].

Limitations of our study reflect a single-center experience. As such, the incidence of Legionnaires' disease in our geographic location may not be representative of other settings. Since we used coded data to determine the number of patients with a discharge diagnosis of pneumonia, we may have underestimated or overestimated the true number of pneumonia cases at our hospital. We combined community and possible hospital-acquired cases to assess the sensitivity of the IDSA/ATS guidelines although the guidelines specifically address community-acquired pneumonia. However, the sensitivity of the recommended testing for Legionella in our 33 community-acquired cases was 58%. Lastly, education programs and hospital-specific diagnostic algorithms in other institutions may promote more widespread *Legionella *testing.

A number of studies have searched for clinical features that are specific for Legionnaires' disease without particular success [8,9]. In endemic areas such as the Eastern USA, where pretest probability is higher [7], our study suggests the possible need to extend urine *Legionella *antigen testing beyond what currently occurs, as suggested by the number of *Legionella *pneumonia cases that did not meet the IDSA/ATS criteria for testing, the proportion of pneumonia cases that are currently being tested for Legionnaires' disease, and the possibility of rising incidence. The best strategy at the present time may be to screen all cases of community-acquired pneumonia requiring hospital admission in endemic locales but the cost-effectiveness of such a strategy first needs to be assessed. Such a testing program will likely identify more *Legionella *pneumonia cases, thereby focusing antimicrobial therapy. Additionally, such testing would better assist public health departments by improving outbreak detection, and it may lead to a better understanding of the epidemiology of Legionnaires' disease and its prevention.

## Conclusions

A diagnostic work-up for *Legionella *(i.e., urine antigen testing or culture of respiratory specimens) is performed in less than half of patients with pneumonia in our endemic region. If *Legionella *testing was curtailed to those recommended in the IDSA/ATS community-acquired pneumonia guidelines, the diagnosis of *Legionella *pneumonia would have been missed in 41% of our cases. Thus, *Legionella *pneumonia may be underdiagnosed and more widespread *Legionella *testing should be considered to better delineate the choice and duration of antimicrobial therapy and to assist in uncovering clusters of *Legionella *in the community or healthcare setting.

## Competing interests

The authors declare that they have no competing interests.

## Authors' contributions

The study idea was developed by LM and BH. Data entry and analysis were performed by ID and BH. BH wrote the first draft of the manuscript which was extensively reviewed by LM and ID. All authors have read and approved the final manuscript.

## Pre-publication history

The pre-publication history for this paper can be accessed here:

http://www.biomedcentral.com/1471-2334/11/237/prepub
